# Functional traits and elemental uptake in urban coastal wetland plants under variable hydrology and edaphic conditions

**DOI:** 10.1093/aobpla/plag006

**Published:** 2026-02-10

**Authors:** Elix Hernandez, Gloria Ortiz-Ramírez, Solimar Pinto-Pacheco, Elvira Cuevas

**Affiliations:** Department of Environmental Sciences, College of Natural Sciences, University of Puerto Rico, Rio Piedras Campus, San Juan 00925-2526, Puerto Rico, USA; Center for Applied Tropical Ecology and Conservation, College of Natural Sciences, University of Puerto Rico, Rio Piedras Campus, San Juan 00925-2526, Puerto Rico, USA; Department of Environmental Sciences, College of Natural Sciences, University of Puerto Rico, Rio Piedras Campus, San Juan 00925-2526, Puerto Rico, USA; Center for Applied Tropical Ecology and Conservation, College of Natural Sciences, University of Puerto Rico, Rio Piedras Campus, San Juan 00925-2526, Puerto Rico, USA; Department of Environmental Sciences, College of Natural Sciences, University of Puerto Rico, Rio Piedras Campus, San Juan 00925-2526, Puerto Rico, USA; Center for Applied Tropical Ecology and Conservation, College of Natural Sciences, University of Puerto Rico, Rio Piedras Campus, San Juan 00925-2526, Puerto Rico, USA; Center for Applied Tropical Ecology and Conservation, College of Natural Sciences, University of Puerto Rico, Rio Piedras Campus, San Juan 00925-2526, Puerto Rico, USA; Department of Biology, College of Natural Sciences, University of Puerto Rico, Rio Piedras Campus, San Juan 00925-2526, Puerto Rico, USA

**Keywords:** coastal urban wetlands, plant functional traits, plant ecophysiology

## Abstract

Coastal wetland plants are adapted to fluctuating and often harsh environmental conditions. In urban wetlands, plant functional groups display a range of physiological and morphological strategies in response to abiotic stress. However, differences amongst functional groups and the coordination between leaf traits, nutrient status, and environmental variation remain poorly understood in these systems. This study evaluates trait–environment relationships in three dominant species—*Acrostichum danaeifolium* (fern), *Dalbergia ecastaphyllum* (nitrogen-fixer shrub), and *Laguncularia racemosa* (halophytic tree)—across contrasting wetland soils and seasonal periods in a tropical urban reserve. We measured leaf gas exchange, specific leaf area (SLA), nutrient content, and photosynthetic nitrogen use efficiency (PNUE) across wet and dry periods on two soils in the Ciénaga Las Cucharillas Natural Reserve, Puerto Rico. Soil bulk density, salinity, and bioavailable nutrients were also quantified. Multivariate analyses (principal component analysis) were used to assess trait covariation. Species differed significantly in morphological and physiological traits. *L. racemosa* exhibited the highest assimilation rates, PNUE, and succulence, consistent with an acquisitive resource-use strategy. In contrast, *A. danaeifolium* showed high SLA and water content but conservative stomatal behaviour and lower PNUE, indicative of a shade-tolerant strategy. *Dalbergia ecastaphyllum* maintained high water-use efficiency during the dry period and exhibited adaptive responses to slightly and moderate saline soils, indicative of a nutrient acquisitive strategy. Soil type influenced elemental availability but had limited effects on photosynthetic rates. Trait differentiation amongst coexisting wetland species reflects contrasting resource-use strategies shaped by both seasonality and soil environment. These findings underscore the functional diversity and adaptive capacity of tropical wetland vegetation under urban and hydrological pressures.

## Teaser

Urban coastal wetland plants can persist under chronic stress, but they do so through different resource-use strategies. By linking leaf traits, nutrient use and plant physiology, we show that coexisting plants adopt different ways of coping with salinity, hydrology and disturbance. This functional diversity supports short-term resilience, but may be vulnerable to future acute disturbances, underscoring the need for trait-informed wetland restoration and management.

## Introduction

Land-use changes for agriculture, urbanization, and industrial development have altered the natural hydrological processes in coastal wetlands, resulting in novel ecosystems characterized by an anthropogenic imprint on hydrological conditions and soil elemental composition (Branoff 2017, Lugo 2009). Present and historical anthropogenic disturbances can lead to temporal and spatial variations in soil elemental composition, water chemical composition, and higher plant diversity of nonindigenous species (Ackerman et al. 2017, Branoff 2017, Newton et al. 2020). Nutrient enrichment from urban sources affects plant community dynamics (Branoff 2018), particularly in wetlands that are naturally nutrient poor (Graham and Mendelssohn 2016, Hayes et al. 2017). Exposure to contaminants such as heavy metals adds a component not seen in unaltered wetlands. Although heavy metals may be present in the soil but not readily available to plants, changes in pH and redox conditions brought about by the spatiotemporal dynamics of wetland hydrology may solubilize the metals, thus rendering them available for plant uptake. Elevated concentrations of heavy metals can cause premature senescence in plants, reduce CO_2_ assimilation, alter soil faunal assemblages, and decrease the productivity of the soil and nutrient cycling through the ecosystems (Nagajyoti et al. 2010). Some plant species, however, can accumulate high levels of heavy metals without being adversely affected (Baker 1981), a fact that makes them potentially useful for phytoremediation purposes.

The intensity and interaction of nutrient enrichment and contaminants exposure vary considerably amongst plant functional groups, and even between closely related species (Chapin et al. 1993). Therefore, evolutionary lineages and ecological strategies should be considered in how these stressors affect ecophysiological processes. In coastal wetlands, baseline hydrological drivers, such as fluctuating salinity and variable nutrient availability, can affect photosynthetic efficiency, stomatal conductance, and elemental uptake amongst coexisting species under the same environmental conditions (Medina et al. 2008, Watzka and Medina 2018). The interaction of these common stressors with additional anthropogenic inputs can generate greater variation in plant performance, reflected in distinct strategies of resource allocation, photosynthetic capacity, water-use efficiency (WUE), and nutrient uptake. Understanding the response of dominant species across different evolutionary lineages remains a question for assessing resilience, productivity, and ecosystem function in urban coastal wetlands under the legacy of historical and ongoing anthropogenic disturbances.

Hydrological conditions affect photosynthesis, light capture, WUE, and nutrient uptake of different plant functional groups (Watzka and Medina 2018). Mangroves are expected to optimize water and salt regulation in saline environments (Medina et al. 2010) and, under high salinity conditions, increase nutrient retranslocation, and leaf respiration (Lugo et al. 2007). Ferns tend to exhibit enhanced shade tolerance and salt tolerance (Lugo et al. 2024, Medina et al. 1990), and nitrogen-fixing shrubs show higher nutrient use-efficiency due to their symbiotic relationship, in nutrient-poor soils (Abbas et al. 2024). These physiological differences contribute to varying roles in ecosystem function and resilience.

This study investigates how variability in soil elemental composition and hydrological conditions affects plant ecophysiological traits (e.g. photosynthetic performance, light capture, WUE, and nutrient uptake) in plants representative of dominant plant functional groups of a tropical urban coastal wetland. We expect that functional differences amongst plant groups would be reflected in measurable ecophysiological traits at the ecosystem scale under a shared mesoclimate. Specifically, (i) *Laguncularia racemosa* (halophytic trees) would exhibit higher exhibit higher instantaneous and intrinsic WUE (higher A/*gs* and lower transpiration rates) as a strategy for coping with saline conditions; (ii) *Acrostichum danaeifolium* (ferns) would display traits associated with shade tolerance and conservative resource use, including lower light-saturated photosynthetic rates and higher specific leaf area (SLA); and (iii) *Dalbergia ecastaphyllum* (nitrogen-fixing shrubs) will demonstrate greater nutrient-use efficiency (higher photosynthetic nitrogen-use efficiency and higher foliar N:P ratios) supported by their symbiotic nitrogen fixation. Collectively, these predictions align with the fast–slow resource-use continuum (Reich, 2014, Wright et al. 2004), where trait variation represents trade-offs between rapid resource acquisition and slow resource conservation strategies. Lastly, we evaluated whether heavy metals-enriched soils alter plant ecophysiological performance. We hypothesize that plants growing in anthropogenically influenced soils will exhibit lower photosynthetic performance [i.e. reduced An, stomatal conductance, and photosynthetic nitrogen use efficiency (PNUE)] because metal accumulation can lead to impairment of photosynthetic machinery and alter foliar elemental composition.

To test these hypotheses, nutrients (N, P, K, Ca, and Mg) and micronutrients (Na, Mn, Cu, Fe, Zn, Pb, Cd, and Al) were determined during the dry and wet seasons in surficial soils and plant leaves in a tropical coastal urban wetland in Puerto Rico. We focused on the available forms of each element, as previous studies have shown these are directly related to plant uptake (Maiti and Jaiswal 2008, Metals 2012). To evaluate the plant response, we measured photosynthetic capacity, WUE, and leaf traits in each species representative of a plant functional group. We discuss the results in the context of the effects of heavily urbanized environments on plant physiology and performance.

## Methods

### Study area

The Ciénaga Las Cucharillas Natural Reserve (18°26′25.27″ N, 66°08′08.39″ W) is an urban coastal wetland located on the western side of the San Juan Bay in the northern metropolitan area of Puerto Rico (Lugo et al. 2011). The site is characterized by a humid climate, with an annual average temperature of 25.2°C and annual precipitation of 1575 mm. Historical human disturbances and water management practices have transformed the wetland from an estuarine mangrove system (Lugo et al. 2011) to a palustrine/estuarine ecosystem (Branoff 2018). Cienaga Las Cucharillas does not follow a simple sea-inland salinity gradient. It is a spatial mosaic of varying salinity, with water table conductivity values ranging spatially from 10 to 35 mS/cm (∼5–22 g kg^−1^ salinity; Hernández et al. 2021). The wetland is part of the Cucharillas drainage basin, located between two large basins that also drain into the bay: the Bayamón and Rio Piedras basins.

The wetland’s hydrology has been modified since colonial times through drainage channels for agricultural use (active until the mid-20th century; Kennaway & Helmer, 2007), the construction of the ‘Malaria Channel’ in the 1940s, which brought a direct flow of freshwater to the wetland from the upper basin (Pumarada-O’Neill and Cruz 1991), and by restricted seawater exchange due to the dyke effect of outflow pumping station at the channel mouth (Webb and Gomez-Gomez 1998). Currently, Las Cucharillas is surrounded by a highly urbanized and industrialized area. Discharges from septic tanks, grey water, illicit intermittent industrial flows and seepages, and atmospheric emissions from power-generating plants affect the wetland, in addition to legacy effects from incidents like the Caribbean Petroleum Corporation explosion in 2009 (U.S. Chemical Safety and Hazard Investigation Board 2015) and clandestine landfills (Subramanian et al. 2018).

### Sampling and analyses

**Figure 1:**
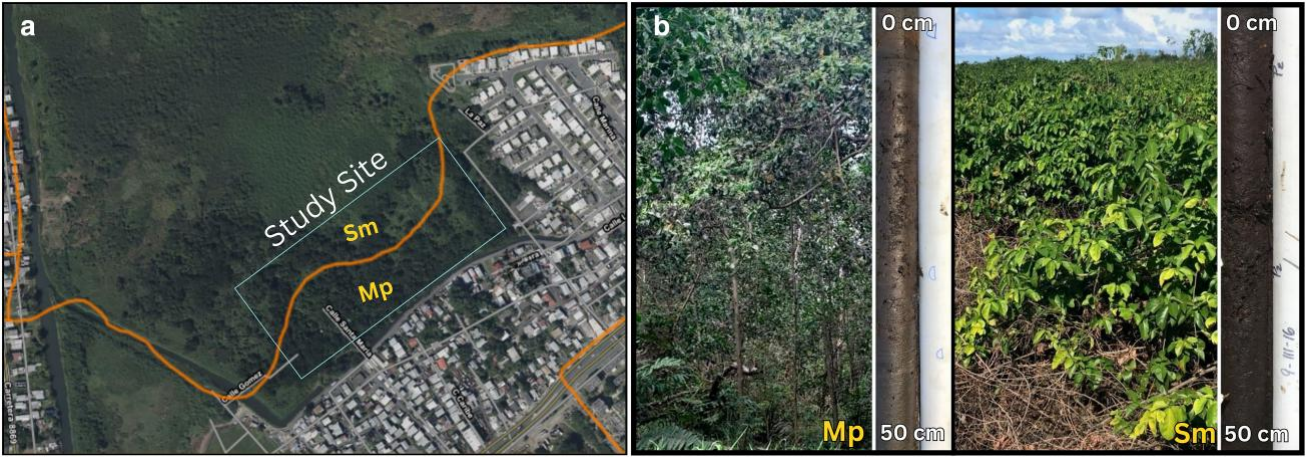
Study site and dominant soil types in Ciénaga Las Cucharillas Natural Reserve, Puerto Rico. Panel a) Aerial imagery showing the study site along the reserve boundary and the distribution of the two dominant soil types: Sm (Saladar muck) and Mp (Martín Peña). Panel b) Representative vegetation and 0–50 cm soil profiles from each soil type. Martin Peña (Mp) soil supports *Laguncularia racemosa* stand with an *Acrostichum danaeifolium* understory in partially shaded areas; the Mp profile shows an organic-rich surface layer (0–5 cm), over mineral sediments embedded. Saladar muck (Sm) supports *Dalbergia ecastaphyllum* shrubland; the soil profile is characterized by black, highly decomposed autochthonous vegetative material. within the study site. Basemap sources: Esri, NASA, NGA, USGS, Airbus.

Within the wetland, we designated four research areas based on distinct soil profiles and hydrological conditions. These correspond to two main soil series, described by the USDA-NRCS: Martin Peña (Mp) and Saladar muck (Sm) soils (Fig. 1). The Martin Peña soil series occurs near urban areas, and is classified as fine, mixed superactive nonacid, isohyperthermic *Humaqueptic Fluvaquents*. They consist of shallow organic material (0–20 cm), over mineral sediments embedded within peat, showing stratigraphical variability associated with past land use disturbance (Griffiths et al. 2021). In contrast, Saladar muck (Sm) soils, located farther from urban areas, are classified as *Typic Haplosaprists* consisting of black, highly decomposed autochthonous organic material that extends to bedrock depth. These soils have a high base and near-neutral pH (7.4 at the surface, decreasing to 6.8 with depth (Soil Survey Staff 2025). Saladar muck (Sm) soils are more organic-rich and less mineralized than Martin Peña (Mp) soils, reflecting limited disturbance and long-term accumulation of decomposed vegetation.

Within each soil type, we selected two plots of 100 m² that differed in water table depth and conductivity. In each plot, three representative wetland species of different plant functional groups were present. One soil core was collected to a depth of 50 cm near each plant functional type using a Russian peat corer, totalling three cores per plot. In total, twelve cores were collected. The cores were placed in halved polyvinyl chloride pipes, wrapped tightly in plastic to prevent desiccation, and stored at 4°C until processed. Fresh subsamples every 10 cm were weighed and subsequently dried at 60°C. The fresh and dry masses were used to calculate gravimetric water content. We calculated bulk density as the quotient between dry mass (MD) and soil volume (V).

Previous sampling in the study area (Weber-Grullon 2016, Yaffar 2017) showed that live root biomass was significantly greater in the 0–10 cm layer compared with deeper layers. Therefore, we selected the upper 10 cm of the soil profile for elemental concentration analyses, as it represents the active root zone. Soil salinity and pH were measured in the Process and Functioning of Tropical Ecosystems Laboratory at the University of Puerto Rico. Salinity (EC) was measured with an EC 300 conductivity meter (YSI, Yellow Springs, OH, USA) using a 1:1 soil-to-water mixture of air-dried soil, and pH was measured on the same slurry using a pH meter (Thermo Fisher Scientific, Waltham, MA, USA). Water levels were measured every 30 min from June 2020 to June 2021 in monitoring wells located in the plot using HOBO water level data loggers (Onset Company, Bourne, MA, USA). The equipment recorded absolute pressure, barometric pressure, and temperature, allowing water level to be determined with a typical error of ±0.1% (±0.4 cm).

Species selection was based on dominance and functional groups’ representation across the hydrological gradient of the study area. Using high-resolution aerial imagery previously acquired with a quadcopter (Hernández et al. 2021), we delineated the spatial extent of the dominant vegetation within each plot and quantified species cover (m^2^). Three species were selected to represent the dominant functional groups present in the wetland: ferns, nitrogen-fixing shrubs, and halophytic trees, based on their relative cover and ecological relevance. The selected species were *A. danaeifolium* Lagnsd. & Fisch (fern), *D. ecastaphyllum* (L.) Taub. (nitrogen-fixer shrub), and *L. racemosa* (L.) C.F. Gaertn. (halophytic tree). Across plots, *Laguncularia* (halophytic tree) dominated areas closer to freshwater inputs, with cover ranging from 92.60% to 2.20%. *Acrostichum* was the only fern recorded, with cover between 0.40% and 7.80%. *Dalbergia* (N-fixer shrub) was restricted to the rich organic soils (Saladar muck, Sm), where it reached 13.30% and 40.4% cover. Additional vegetation included grasses and herbs, each contributing less than 10% cover and excluded from the ecophysiological analyses. One species from each functional group was selected to ensure representation of distinct ecological strategies across the hydrological gradient.

*Acrostichum* (fern) is an herbaceous, rhizomatous species from the fern family (Pteridaceae) common in brackish swamps, tolerant to soil saline conditions up to 30 g kg^−1^, but requires freshwater for establishment. High levels of soil salinity and light conditions (understory vs. open canopy) will produce changes in plant size and density (Medina et al. 1990). *Dalbergia* (N-fixer shrub) is a decumbent shrub with branches reaching 1–5 m in length. It is branched, with alternate leaves composed of one or exceptionally three ovate, coriaceous leaflets (Acevedo-Rodríguez 2005). *Dalbergia* (N-fixer shrub) grows in a range of ecosystems from nonforested areas, freshwater wetlands, to dunes. *Dalbergia* (N-fixer shrub) forms monospecific stands, helping soil retention and stabilization (Pereira and Mata 2014). It develops a taproot and a lateral root system with nitrogen-fixing root nodules (Rhizobium) growing on the shallow roots (Saur et al. 2000). *Laguncularia* (halophytic tree) is a salt-tolerant species, with succulence and salt-secreting adaptations being the main mechanisms, allowing tolerance for a wide range of soil salinity (Parida and Jha 2010). Recent studies have confirmed this species as having a large level of plasticity to environmental factors (Da Souza et al. 2014).

Rainfall in the northern Caribbean has a bimodal pattern (Taylor et al. 2002). Puerto Rico has two wet periods: a peak in July–November, another in May–June, and a dry season from December to April (Torres-Valcárcel et al. 2014). To account for temporal variability, three to five adult, sun-exposed, fully expanded leaves (up to a 2.5 m height) were collected from each of three individuals per plot and per species during four sampling times (during wet and dry periods). This yielded ∼36–60 leaves per species per sampling period or 144–240 leaves per species annually. Across the three species, a total of 400–700 leaves were collected over a year. From this pool, 204 leaves (75 leaves *Acrostichum*, 34 *Dalbergia*, and 95 from *Laguncularia*) were transported in a cooler to prevent water loss and used to measure leaf area, length, and width with an LI-3100C (LI-COR, Lincoln, NE, USA) area meter. Fresh leaf mass was determined before area measurements. All leaves of each species were dried in a forced-air oven at 60°C until constant mass for dry mass determination. In the case of *Acrostichum*, individual leaflets were measured due to their compound fronds. SLA (the area of a fresh leaf divided by its oven-dry mass), leaf water content, and succulence were calculated for each sample.

### Soil sample preparation and analysis

Soil samples for exchangeable element analysis (Na, Mg, Al, K, Ca, P, Mn, Fe, Zn, Cu, Cd, and Pb) were sent to the ICP-MS Metals Lab at the Department of Geology and Geophysics, University of Utah. A leaching procedure was performed in which 200 mg of air-dried soil was weighed and added, 4 ml of 1 molar ammonium acetate buffered to pH 7, stirred, and left overnight. Samples were centrifuged at 5000 rpm for 5 min, and the supernatant was collected for analysis. This procedure allows the extraction of water-soluble elements and the desorption of cations from soil fine particles. Samples were run in an Agilent 7500ce quadrupole inductively coupled plasma mass spectrometer (Agilent, Santa Clara, CA, USA). The Standard Reference Material 1643e (National Institute of Standards and Technology, Gaithersburg, MD, USA) was used (Goodman et al. 2019). Results were expressed in mg/kg and converted to mmol/kg based on the molar mass of each element.

### Plant sample preparation and measurements

Elemental analysis of Na, Mg, Al, K, Ca, P, Mn, Fe, Zn, Cu, Cd, and Pb in leaf samples was conducted at the ICP-MS Metals Lab at the Department of Geology and Geophysics, University of Utah. Leaf tissues (0.5 g dry mass) in porcelain crucibles were burned in a muffle furnace for 8 h at 550°C, after cooling, the ashes were weighed and dissolved in 4 ml of 5% HNO^3^. Crucibles were rinsed with 5% HNO^3^ and added to a final volume of 10 ml of 5% HNO^3^. Samples were centrifuged at 3000 rpm for 5 min, and an aliquot of 0.5 ml of the supernatant was diluted to 10 ml. Samples were analysed in an Agilent 7500ce quadrupole inductively coupled plasma mass spectrometer (Agilent, Santa Clara, CA, USA). The Standard Reference Material 1643e (National Institute of Standards and Technology, Gaithersburg, MD, USA) was used (Bitter et al. 2020). Plant total elements were expressed in mg/kg and converted to mmol/kg using the molar mass of each element.

### Light response curves

Fully expanded, sun-exposed leaves were sampled from three individuals, one from each plot from the species: *Laguncularia* (halophytic tree), *Acrostichum* (fern), and *Dalbergia* (N-fixer shrub). Light response curves were performed to measure variations in PAR, air and leaf temperature, air humidity, and CO_2_ concentration to estimate net carbon assimilation (*A_net_*), stomatal conductance (*g_s_*), transpiration rate (*E*), WUE, and intercellular CO₂ concentration (*ci*). These measurements were conducted using an infrared gas analyser (CIRAS3, PP Systems, Amesbury, MA, USA), with an integrated LED (PLC3) light unit source over 10 levels of light intensities (PAR): 2000, 1500, 1000, 800, 500, 250, 100, 50, 25, and 0 µmol photons m⁻² s⁻¹ at a constant flow rate of 300 cm^3^ min^−1^, starting at ambient irradiance, to highest values and switching to lowest values with 2–7 min intervals. Ambient temperature averaged 33°C and leaf temperature averaged 34°C. The cuvette temperature was set to follow leaf temperature. Measurements were made at a controlled 400 µmol mol⁻¹ CO_2_ concentration. The light response curves were fitted to the data using JMP Pro (SAS Institute Inc., Cary, NC, USA) using the equation by Prioul and Chartier (1977):


$$\begin{array}{r} P=\left(\frac{(\Phi \cdot \mathrm{PA}R_{i}+A_{\mathrm{sat}})-\sqrt{(\Phi \cdot \mathrm{PA}R_{i}+A_{\mathrm{sat}})2-4\cdot \theta \cdot \Phi \cdot \mathrm{PA}R_{i}\cdot A_{\mathrm{sat}}}}{2\cdot \theta }\right)-R_{d} \end{array}$$

where maximum photosynthetic capacity at saturating light intensity (*A*_sat_), apparent quantum yield (ϕ), dark respiration rate (*R_d_*), and curve convexity (*θ*) were calculated, based on the incident photosynthetically active radiation (PAR*_i_*, µmol photons m⁻² s⁻¹).

### Plant photosynthetic assimilation under field conditions

Three to five adult leaves with full sun exposure were selected from each species at each plot. Light conditions were controlled with the PLC3 LED light unit (RGBW) of the CIRAS 3 IRGA (PP Systems, Amesbury, MA, USA) and light intensity was set to 800 µmol m^−2^ s^−1^. This irradiance was chosen for two reasons: (i) it represented the average mid-morning photosynthetic photon flux density (PPFD) measured *in situ* (7:00–11:00 a.m.) using data from a nearby weather station during the sampling period and (ii) light response curves showed photosynthesis approaching saturation between 600 and 1000 µmol m⁻² s⁻¹ across species. Hence, 800 µmol m⁻² s⁻¹ provided near-saturating conditions suitable for standardized comparisons between species. All measurements were done over a period of 2 days before noon (7:00–11:00 a.m.). Sampling was carried out in June 2020 and February 2021.

### Statistical analysis

To evaluate differences in soil and leaves elemental concentrations, we used linear mixed-effects models with soil type (Martin Peña, *Mp* and Saladar muck, *Sm*) and period (wet and dry) as fixed effects, and plot as a random effect to account for spatial replication and uneven sample sizes. Foliar morphological traits (leaf area, dry mass, SLA, leaf water content, and succulence) we used one-way ANOVA amongst species. Photosynthetic traits were analysed using three-way ANOVA with plant functional types, period, and soil types as fixed factors. To explore multivariate patterns in foliar traits, leaf elemental composition, and species, a principal component analysis (PCA) was performed using centred variables. All statistical analyses were conducted using JMP Pro (SAS Institute Inc., Cary, NC, USA).

## Results

### Climate conditions

**Figure 2:**
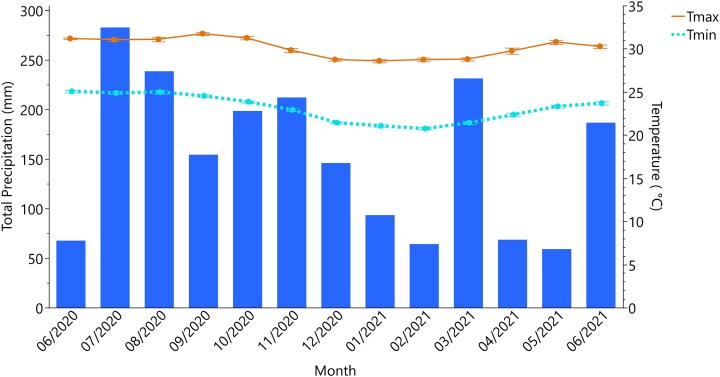
Climate conditions during the study period (June 2020–June 2021) near Cienaga Las Cucharillas Natural Reserve, Puerto Rico. Blue bars show monthly total precipitation (mm). Lines show monthly mean air temperatures: maximum (*T*_max_, orange) and minimum (*T*_min_, sky blue dashed), with error bars indicating standard error (*n* = 30–31 per month).

Rainfall exhibited the characteristic bimodal pattern of the island with two distinct wet periods separated by intervening dry periods (Fig. 2; Taylor et al. 2002, Torres-Valcárcel et al. 2014). Notably, during the dry period of 2021, an anomalous precipitation event occurred on March 21, when 168 mm of precipitation was recorded in a single day, substantially exceeding the cumulative 63 mm recorded over the remainder of the month. This event temporarily inundated the wetland ecosystem. Precipitation in June demonstrated interannual variability, with the month alternating between wet and dry conditions depending on the year. The island's bimodal climatic pattern was also evident in the monthly temperature trends (Fig. 2), where cooler temperatures coincided with the dry period and elevated temperatures aligned with the wet period.

### Soil abiotic factors

**Figure 3:**
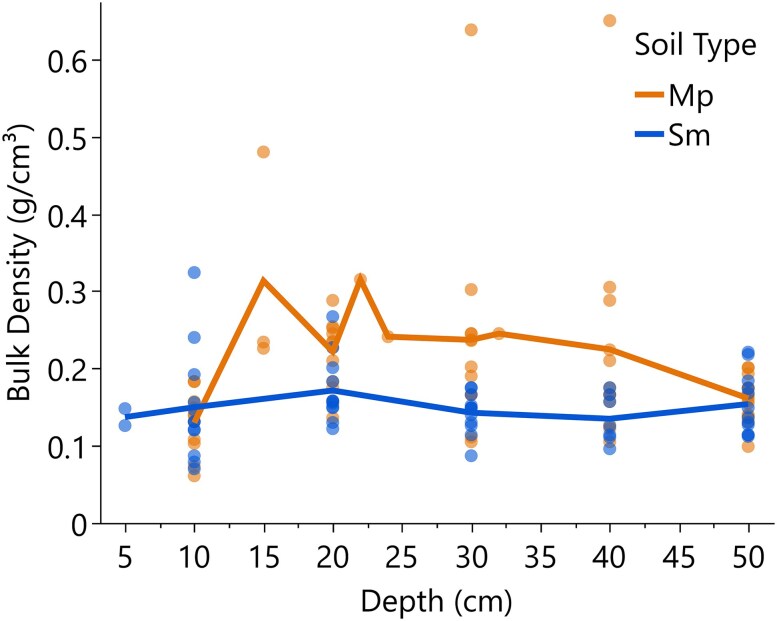
Bulk density profiles (g cm^3^) of Martin Peña (Mp) and Saladar muck (Sm) soils measured at depths from 5 to 50 cm. Points represent individual measurements, and lines indicate mean bulk density.

Soil salinity averaged 5 g kg^−1^ during the dry period and increased to 7.1 g kg^−1^ in the wet period (Table 1). During the wet period, water inputs originated not only from rainfall but also from tidal influences affecting the phreatic zone, particularly under waterlogging conditions, soil salinity during the dry period was temporarily lowered due to the atypical single-day precipitation event. Average bulk density ranged from 0.06 to 0.65 g cm⁻³ across sites and depths. Bulk density differed significantly between soil types (*P* = 0.002) with Martin Peña (*Mp*) soil (mean 0.20 ± 0.10 g cm⁻³) exhibiting a slightly higher mean bulk density than Saladar muck (*Sm*) (0.15 ± 0.06 g cm⁻³) across all depths (Table 1, Fig. 3).

**Table 1:** Edaphic abiotic factors were measured in four study plots within the Ciénaga Las Cucharillas Natural Reserve during the wet and dry periods.

Soil type (USDA class)	Martin Peña (Mp)				Saladar Muck (Sm)			
Plots	Plot 3		Plot 5		Plot 6		Plot 10	
Period	*Wet*	*Dry*	*Wet*	*Dry*	*Wet*	*Dry*	*Wet*	*Dry*
pH (1:1)	4.76	6.10	4.64	5.20	4.60	5.60	4.80	6.10
Soil salinity (1:1) g kg^−1^	3.4 ± 2.0	4 ± 0.4	2.8 ± 1.0	12 ± 2.0	2.4 ± 2.0	11 ± 5.0	2.1 ± 2.0	7 ± 1.0
Bulk density 10 cm (g/cm^3^)	0.11 ± 0.05	0.16 ± 0.03	0.10 ± 0.07	0.12 ± 0.05	0.17 ± 0.06	0.13 ± 0.06	0.20 ± 0.10	0.10 ± 0.03
Bulk density 0–50 cm (g/cm^3^)	0.16 ± 0.06		0.21 ± 0.13		0.16 ± 0.04		0.14 ± 0.05	
SOM %	35.30 ± 15.00		31.30 ± 7.00		36.70 ± 11.00		32.90 ± 15.00	

Plots 3 and 5 were in Martín Peña (Mp) soils, while Plots 6 and 10 are located in Saladar Muck (Sm) soils. Values represent mean ± standard deviation (*n* = 3 per plot).

### Spatial and temporal elemental variability in soils

For the active root zone (in the first 10 cm), there were no significant differences amongst soil samples collected near each functional plant type within the same plot. Therefore, all species in a given plot experienced similar soil element availability conditions. Thus, differences in leaf element content are attributed to plant physiology rather than site variation. In the exchangeable elements analysis, there were significant differences between soil types for Al, and significant seasonal differences in available Al, P, Zn, and Pb (Table 2). Soil profiles down to 50 cm revealed differentiation in exchangeable element composition between Martin Peña (Mp) and Saladar muck (Sm) soils, reflecting distinct chemical characteristics (Supplementary Figures S1–4). The concentrations of Na and Mg were generally higher in Martin Peña (Mp) soils, particularly in the upper 15 cm (∼800–1200 mmol/kg for Na and ∼200–300 mmol/kg for Mg), indicating greater influence from salinity intrusion or anthropogenic inputs compared to Saladar muck (Sm) soils. The soil concentration of available Al increased with depth in Saladar muck (Sm) soils, contrasting with more stable values throughout the Martin Peña (Mp) soil profile. Transition metals, such as Zn, Pb, Cd, and Cu, showed higher concentrations near the surface layers of Martin Peña (Mp) soils (Zn ∼0.3–0.5 mmol/kg, Pb ∼200–300 µmol/kg, Cd ∼2 µmol/kg, and Cu ∼20 µmol/kg), suggesting anthropogenic pollution from urban and industrial sources. In contrast, P and K concentrations remained relatively low and stable in both soil types, consistent with nutrient-limited conditions typical of wetland ecosystems. These depth-wise variations underline the strong spatial and vertical heterogeneity in soil chemistry, potentially influencing plant nutrient uptake and tolerance to metal toxicity in urban coastal wetlands.

**Table 2:** Elements (mean ± standard deviation) in the top 10 cm of soil during rainy and dry periods.

Elements	Martin Peña soil type		Saladar Muck soil type		Statistical significance	
	Wet July 20 (*n* = 7)	Dry May 21 (*n* = 6)	Wet Sept 20 (*n* = 8)	Dry May 21 (*n* = 6)	Soil type	Period
Na	777.5 ± 301.6	513.6 ± 182.8	549.5 ± 278.5	549.3 ± 268.6	*F*_1, 2_ = 0.29ns	*F*_1,24_ = 1.63ns
K	13.30 ± 3.93	13.0 ± 6.6	11.3 ± 2.19	13.0 ± 3.38	*F*_1,2_ = 0.83ns	*F*_1,24_ = 0.39ns
Ca	162.1 ± 49.07	186.0 ± 43.69	171.2 ± 55.21	266.2 ± 66.69	*F*_1,2_ = 1.93ns	*F*_1,24_ = 8.53*
Mg	187.2 ± 50.20	142.9 ± 35.91	160.1 ± 62.21	174.0 ± 53.82	*F*_1,2_ = 0.02ns	*F*_1,24_ = −0.70ns
Al	0.79 ± 0.42	0.22 ± 0.07	0.54 ± 0.24	0.23 ± 0.07	*F*_1,26_ = 14.7***	*F*_1,26_ = 28.6***
P	0.30 ± 0.20	0.45 ± 0.17	0.30 ± 0.33	0.61 ± 0.20	*F*_1,2_ = 0.13ns	*F*_1,24_ = 8.92**
Mn	0.71 ± 0.39	0.52 ± 0.23	0.65 ± 0.68	0.49 ± 0.11	*F*_1,2_ = 0.02ns	*F*_1,24_ = 1.95ns
Fe	1.05 ± 1.18	0.81 ± 0.64	0.50 ± 0.31	0.59 ± 0.11	*F*_1,2_ = 2.96ns	*F*_1,24_ = 0.14ns
Zn	0.53 ± 0.30	0.31 ± 0.20	0.19 ± 0.16	0.09 ± 0.030	*F*_1,2_ = 1.29ns	*F*_1,24_ = 11*
Cu	35.77 ± 42.15	71.63 ± 91.22	6.31 ± 5.91	16.73 ± 3.8	*F*_1,2_ = 1.24ns	*F*_1,24_ = 1.82ns
Cd	0.85 ± 0.59	0.62 ± 0.46	0.64 ± 0.64	0.19 ± 0.08	*F*_1,2_ = 0.36ns	*F*_1,24_ = 6.03*
Pb	62.68 ± 55.12	16.13 ± 33.30	13.38 ± 14.82	1.05 ± 0.63	*F*_1,2_ = 3.24ns	*F*_1,24_ = 6.08*

Macronutrients (Na, K, Ca, Mg, Al, P, Mn, Fe, and Zn) are expressed in mmol/kg and trace metals (Cu, Cd, and Pb) are expressed in µmol/kg. Sample sizes are shown for each soil type per period group (*n* = 6–8). Statistical results reported as *F*_df1,df2_, with significance codes: ***, *P* < 0.001; **, *P* < 0.01; *, *P* < 0.05; ns non-significant.

The analysis of soil element availability in the upper 10 cm layer revealed significant spatial and temporal variability in the wetland soils studied (Table 2). Notably, Al, P, Zn, and Pb exhibited statistically significant variations. Martín Peña (Mp) soils showed markedly higher concentrations of Al, especially during the wet period (Table 2) indicating greater anthropogenic influence compared to Saladar muck (Sm) soils. Additionally, P and Ca content tended to be higher during the dry season, although these increases were not statistically significant (Table 2), potentially due to reduced waterlogging. Conversely, Al availability increased during the wet period, likely reflecting increased solubility under anoxic conditions. Between soils or sampling dates, K and Na did not differ significantly, suggesting consistent availability independent of environmental conditions. The Pb availability was particularly higher in Martin Peña (Mp) soils, indicating greater anthropogenic metal inputs.

### Spatial and temporal elemental variability between species

**Figure 4:**
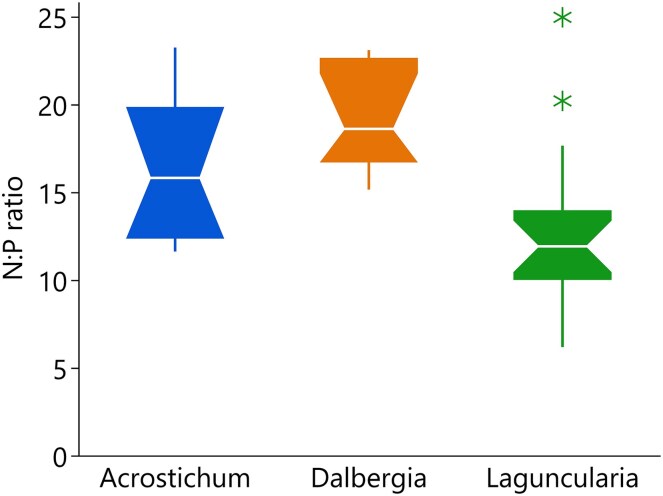
Boxplots showing N:P ratios for the three studied species. Notches represent ∼95% confidence intervals for median N:P values.

Leaf elemental concentrations varied significantly amongst the studied species, indicating distinct physiological strategies and ecological niche differentiation (Table 3). *Acrostichum* (fern) had the highest Na and Ca concentrations. *Laguncularia* (halophytic tree) showed elevated P, Mg, and Fe levels, along with high Pb accumulation. In contrast, *Dalbergia* (n-fixer shrub) exhibited significantly lower Na concentrations, while accumulating higher levels of zinc amongst the three species. In none of the species, K content was higher than Na, and the K/Na ratios were as follow *Laguncularia* (halophytic tree): 0.13 ± 0.06, *Acrostichum* (fern): 0.18 ± 0.18, and *Dalbergia* (N-fixer shrub): 1.45 ± 1.20. Species-level differences in foliar N:P ratios were noted with *Dalbergia* (N-fixer shrub) and *Acrostichum* (fern) exhibiting the highest N:P values (Fig. 4), albeit *Acrostichum* (fern) slightly lower, and *Laguncularia* (halophytic tree) displaying lower N:P values, indicating contrasting nutrient-use strategies amongst the three species.

**Table 3:** Leaf element concentrations (mean ± standard deviation) for *Acrostichum* (n = 19), *Dalbergia* (n = 14), and *Laguncularia* (n = 28).

Element				Statistical significance					
	Concentrations			Soil Type		Period			Species
	*Acrostichum* (N = 19)	*Dalbergia* (N = 14)	*Laguncularia* (N = 28)	*Acrostichum*	*Laguncularia*	*Acrostichum*	*Dalbergia*	*Laguncularia*	
N	31.03 ± 6.40	26 ± 3	1 ± 3	*F*_1,2_ = 0.78 ns	*F*_1,2_ = 0.48ns	*F*_1,14_ = 0.04ns	*F*_1,6_ = 41.1***	*F*_1,13_ = 10.41**	*F*_2,31_ = 57.9***
Na	440.50 ± 157.09	68.9 ± 120.42	323.9 ± 138.60	*F*_1,2_ = 7.06ns	*F*_1,2_ = 2.44 ns	*F*_1,15_ = 5.44*	*F*_1,10_ = 44.5***	*F*_1,24_ = 0.71ns	*F*_2,57_ = 31.5***
Mg	107.3 ± 27.63	79.2 ± 17.94	170.3 ± 28.49	*F*_1,2_ = 0.12ns	*F*_1,1_ = 0.57ns	*F*_1,14_ = 0.08ns	*F*_1,11_ = 148***	*F*_1,24_ = 0.31ns	*F*_2,25_ = 81***
Al	1.77 ± 1.29	4.97 ± 13.46	1.30 ± 1.13	*F*_1,2_ = 1.6ns	*F*_1,2_ = 2.32ns	*F*_1,14_ = 1.4ns	*F*_1,10_ = 1.98ns	*F*_1,24_ = 0.93ns	F_2,49_ = 1.50ns
K	64.3 ± 26.02	36.0 ± 4.54	35.9 ± 8.04	*F*_1,2_ = 0.75ns	*F*_1,2_ = 0.37ns	*F*_1,14_ = 0.56ns	*F*_1,10_ = 0.001ns	*F*_1,24_ = 0.15ns	*F*_2,57_ = 11.4***
Ca	376.9 ± 179.04	187.5 ± 64.34	259.0 ± 106.5	*F*_1,2_ = 0.12ns	*F*_1,2_ = 0.08ns	*F*_1,14_ = 0.27ns	*F*_1,11_ = 12**	*F*_1,24_ = 8.09**	*F*_2,53_ = 35.3**
P	161.47 ± 122.50	206.63 ± 120.76	489.48 ± 199.72	*F*_1,2_ = 0.26ns	*F*_1,2_ = 0.19ns	*F*_1,14_ = 7.01*	*F*_1,10_ = 3.42ns	*F*_1,24_ = 16.5**	*F*_2,56_ = 26.7***
Mn	1.15 ± 0.47	6.80 ± 2.47	1.00 ± 0.39	*F*_1,2_ = 0.20ns	*F*_1,2_ = 0.19ns	*F*_1,14_ = 0.02ns	*F*_1,10_ = 16.3**	*F*_1,24_ = 0.26ns	*F*_2,54_ = 160***
Fe	3.84 ± 4.38	6.47 ± 13.54	11.28 ± 7.01	*F*_1,2_ = 1.52ns	*F*_1,2_ = 0.84ns	*F*_1,14_ = 1.2ns	*F*_1,11_ = 2.96ns	*F*_1,24_ = 0.40ns	*F*_2,47_ = 5.5*
Zn	0.49 ± 0.28	1.15 ± 0.41	0.52 ± 0.32	*F*_1,2_ = 0.51ns	*F*_1,2_ = 1.47ns	*F*_1,14_ = 3.22ns	*F*_1,11_ = 0.47ns	*F*_1,24_ = 0.24ns	*F*_2,56_ = 50.6***
Cu	174.35 ± 112.49	112.40 ± 65.80	105.21 ± 36.59	*F*_1,2_ = 0.26ns	*F*_1,2_ = 2.8 ns	*F*_1,14_ = 0.01ns	*F*_1,10_ = 0.42ns	*F*_1,24_ = 0.50ns	*F*_2,38_ = 10.02**
Cd	1.00 ± 0.78	1.49 ± 0.97	1.88 ± 2.36	*F*_1,6_ = 1ns	*F*_1,2_ = 1.5ns	na	na	na	*F*_2,15_ = 9.4*
Pb	2.00 ± 3.43	3.36 ± 7.72	8.57 ± 6.90	*F*_1,1_ = 226*	*F*_1,2_ = 1.87ns	*F*_1,15_ = 1.8ns	*F*_1,11_ = 1.24ns	*F*_1,24_ = 9.65*	*F*_2,57_ = 5.8*

Nitrogen is expressed in g kg^−1^. Macronutrients (Na, K, Ca, Mg, Al, P, Mn, Fe, and Zn) are expressed in mmol kg^−1^ and trace metals (Cu, Cd, and Pb) are expressed in µmol kg^−1^. Statistical results are reported as *F*_df1,df2_, with significance codes: ***, *P* < 0.001; **, *P* < 0.01; *, *P* < 0.05; ns non-significant. ‘na’ denotes concentrations below the instrument detection limit and not analysed statistically.

A PCA of leaf element composition by species revealed that the first principal component (PC1) accounted for 25% of the variance and the second component (PC2) accounted for 29.9%, together explaining 58.9% of the total variance (Fig. 7). Mg, Fe, and Ca were strongly aligned with PC1, while Zn and Mn were aligned with PC2. The PCA revealed distinct species-specific clustering patterns: *Dalbergia* (n-fixer shrub) formed a separate cluster, *Acrostichum* (fern) and *Laguncularia* (halophytic tree) showed partial overlap, suggesting a degree of similarity in their foliar elemental composition.

Leaf morphological traits varied significantly amongst the studied species (Table 4). Average leaf area followed the sequence *Acrostichum* (fern) *> Dalbergia* (N-fixer shrub) *>*  *Laguncularia* (halophytic tree) with values of 49.42, 45.12, and 27.35 cm², respectively. *Dalbergia* (N-fixer shrub) leaves exhibited the highest leaf dry mass (∼0.44 g) compared to *Acrostichum* (fern) (∼0.32 g) and *Laguncularia* (halophytic tree) (∼0.34 g). Consequently, *Acrostichum* (fern) displayed the highest SLA (163.02 ± 50.61 cm² g⁻¹), whereas *Laguncularia* (halophytic tree) had the lowest (84.68 ± 22.14 cm² g⁻¹), and *Dalbergia* (N-fixer shrub) had intermediate values (110.15 ± 38.09 cm² g⁻¹). Leaf water content was significantly lower in *Dalbergia* (N-fixer shrub) (∼58%) than in *Acrostichum* (fern) (∼75%) or *Laguncularia* (halophytic tree) (∼70%). Succulence, defined as the amount of water stored per unit leaf area was highest in *Laguncularia* (halophytic tree) (∼289 g m⁻²), lowest in *Dalbergia* (N-fixer shrub) (∼137 g m⁻²), and *Acrostichum* (fern) exhibiting intermediate (∼199 g m⁻²) values.

**Table 4:** Leaf morphological traits for the three studied species. Values represent means ± standard deviations.

Leaf traits	*Acrostichum (fern)*	*Dalbergia (N-fixer shrub)*	*Laguncularia (halophytic tree)*
Leaf area (cm^2^)	49.42 ± 15.99 a	45.12 ± 15.97 b	27.35 ± 7.71 c
Dry mass (g)	0.32 ± 0.14 b	0.44 ± 0.19 a	0.34 ± 0.12 b
SLA (cm^2^/g)	163.02 ± 50.61 a	110.15 ± 38.09 b	84.68 ± 22.14 c
Leaf water content (%)	74.85 ± 4.91 a	58.03 ± 6.98 c	69.77 ± 5.06 b
Succulence (g/m^2^)	199.32 ± 38.71 b	137.35 ± 30.60 c	289.62 ± 51.77 a

Different letters denote significant differences amongst species within each trait (one-way ANOVA, *P* < 0.05, post-hoc test).

**Figure 5:**
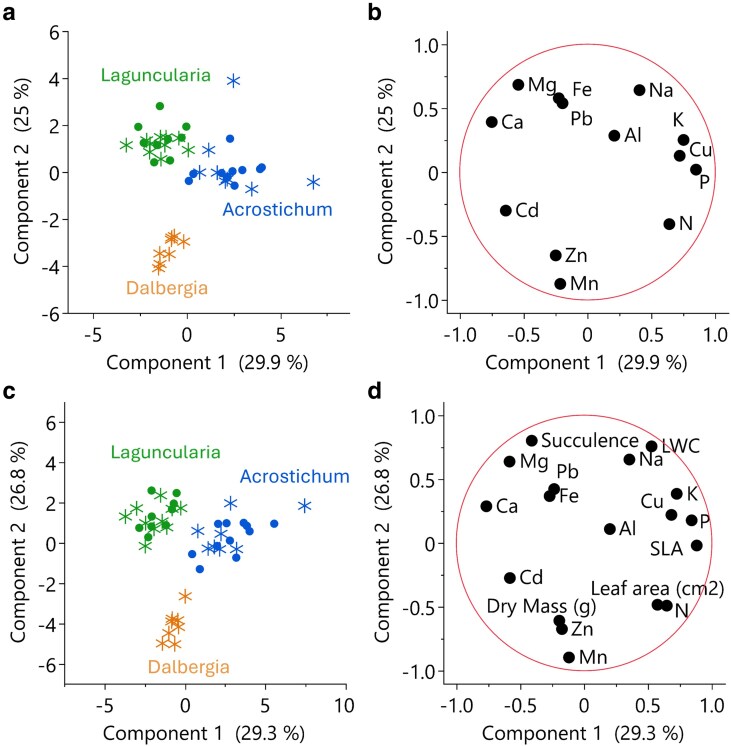
Principal component analysis (PCA) of leaf element concentrations for the three species. a) PCA scores for element concentrations showing separation amongst the halophytic tree *Laguncularia racemosa* (green), the fern *Acrostichum danaeifolium* (blue), and the N-fixer shrub *Dalbergia ecastaphyllum* (orange). b) Corresponding PCA loadings plot showing element concentration vectors. c) PCA scores of leaf morphological traits and elements, showing species-level clustering. d) Corresponding loading plots for leaf traits and elements. In score plots (a, c) symbol type denotes soil type: Saladar muck (Sm, asterisks) and Martin Peña (Mp, circles).

When analysing relations between leaf traits and elemental concentrations, a PCA (Fig. 5) showed that PC1 accounted for 28.6% of the total variance associated with high succulence and elevated concentrations of Na, Mg, and Ca. PC2 explained 29.3% of the variance. Together, PC1 and PC2 explained 57.9% of the total variance. *Dalbergia* (N-fixer shrub) scored high on PC2, reflecting its greater leaf mass and accumulation of Mn, and Zn, and suggesting the presence of thicker, denser leaves capable of sequestering these metals. In contrast, *Acrostichum* (fern) and *Laguncularia* (halophytic tree) clustered towards high PC1 values, indicative of greater succulence and cation accumulation.

### Light response curves

**Figure 6:**
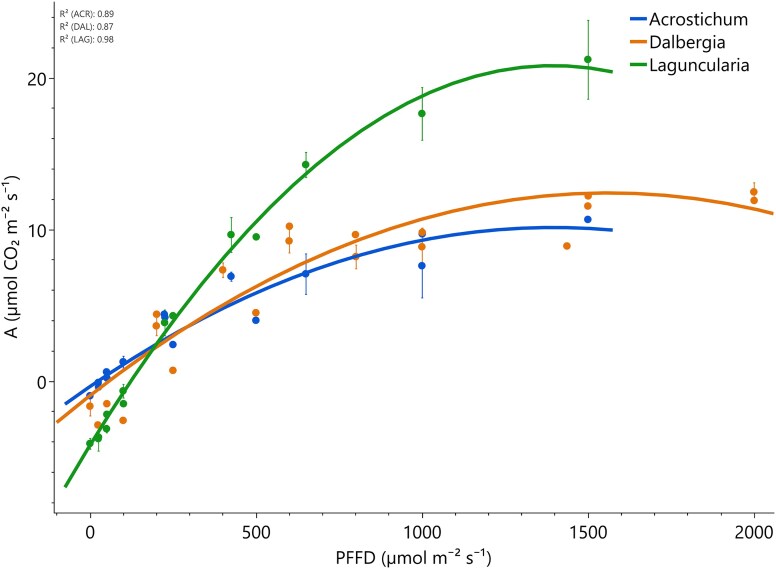
Light response curves of photosynthetic assimilation (a) as a function of PPFD for the species studied. Each curve represents a nonlinear fit to data collected from three replicate individuals per species. Error bars indicate the SEM. Coefficient of determination (*R*²) values for each species are shown in the top left.

Photosynthetic light-response parameters differed between species (Fig. 6). *Laguncularia* (halophytic tree) exhibited the highest light-saturated photosynthetic rate and apparent quantum yield, but also had the highest dark respiration rate and light compensation point. *Acrostichum* (fern) displayed the lowest *A*_sat_, the lowest *ϕ*, the lowest LCP, and the lowest *R_d_*, consistent with a shade-tolerant strategy. *Dalbergia* (N-fixer shrub) showed intermediate performance across most parameters (e.g. LCP ∼ 63, *R_d_* ∼ 2.0). All species had similar curvature values (*θ* ∼ 0.53–0.77). Overall, *Laguncularia* (halophytic tree) appeared well adapted to high light environments with high photosynthetic capacity, *Dalbergia* (N-fixer shrub) demonstrated moderate photosynthetic performance with greater parameter variability, while *Acrostichum* (fern) was characterized by shade-adapted physiology and low light requirement.

### Field photosynthesis measurements

**Figure 7:**
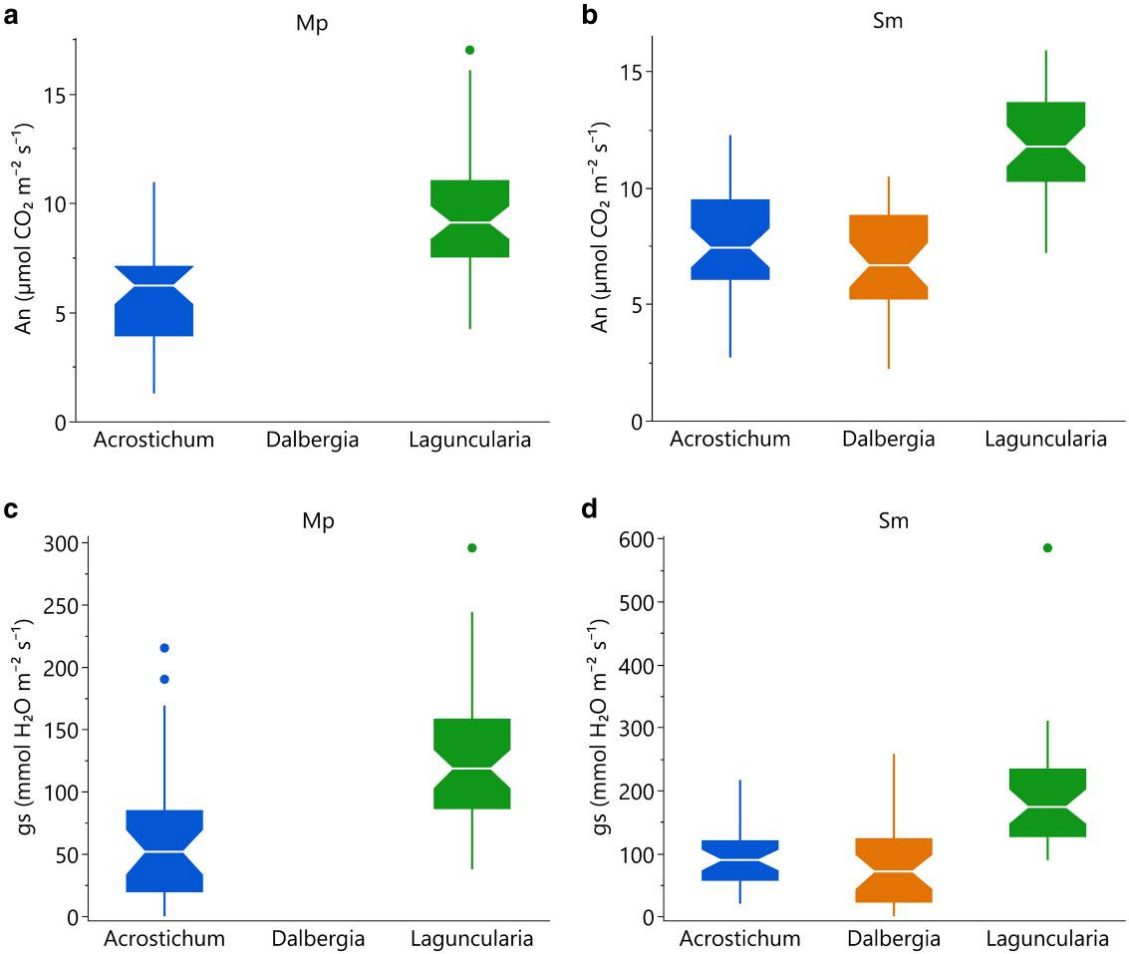
Mean ± standard error net photosynthetic rate (*A*_net_; panels (a, b) and *g_s_* (panels (c, d) for the three species studied measured in two soil types: Martin Peña (Mp; a and c) and Saladar muck (Sm; b and d). Values represent species means pooled across sampling periods. Notches represent ∼95% confidence intervals for median values.

**Figure 8:**
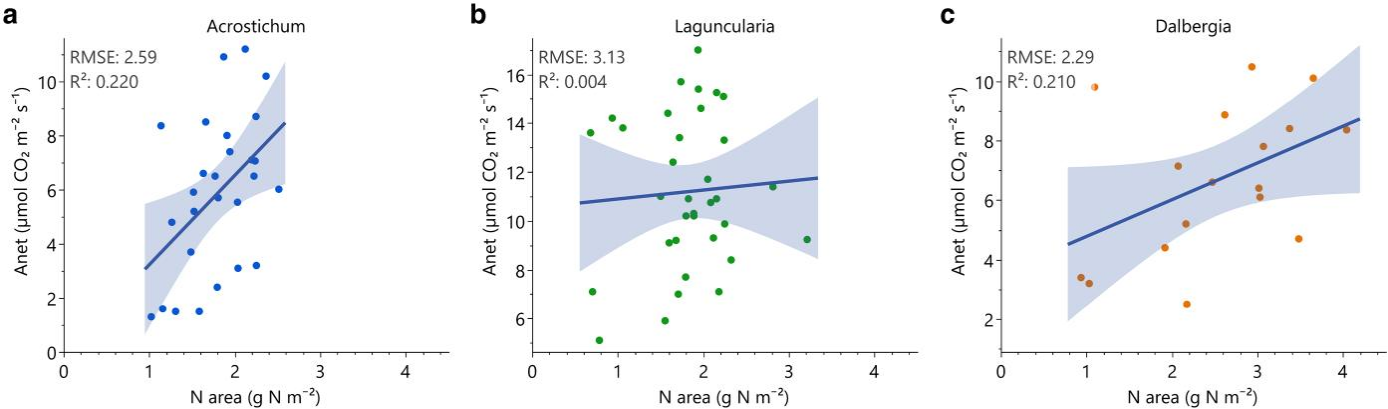
Linear relationships between foliar N per unit area (*N*_area_) and net photosynthes*i*s (A_net_) for *Acrostichum* (a), *Dalbergia* (b), and *Laguncularia* (c). Solid lines represent linear regressions with 95% confidence bands. RMSE and *R*^2^ values are shown for each species.

Field measurements indicated significant variations in photosynthetic performance across species, hydrological periods, and soil types (Table 5). *Acrostichum* (fern) exhibited notably higher assimilation rates (*A_net_*) in Saladar muck (Sm) soils compared to Martin Peña (Mp) soils, reflecting significantly more favourable physiological conditions in the former soil type (Fig. 7). Period-related differences were also evident, with stomatal conductance (*g_s_*) significantly increasing during the wet period, promoting greater transpiration but reducing WUE. Similarly, *Laguncularia* (halophytic tree) exhibited significantly higher *A_net_*, *g_s_*, and *E* during the wet period in Sm soils. *Dalbergia* (N-fixer shrub) displayed a pronounced seasonal response in *A_n_* and WUE. Photosynthetic nitrogen use efficiency (PNUE) defined as the ratio of photosynthetic capacity to leaf N content (reported as µmol CO₂ m⁻² s⁻¹ per g N kg⁻¹) was significantly higher in *Laguncularia* (halophytic tree). Across species, when analysing the correlation between An and leaf nitrogen (per unit area), we found a positive correlation suggesting that higher *N*_area_ is associated with greater photosynthetic capacity in *Acrostichum* (fern), *Dalbergia* (N-fixer shrub) exhibits a moderate positive association between leaf *N*_area_ and photosynthetic capacity. However, in *Laguncularia* (halophytic tree) despite high *A*_net_ rates, no meaningful relationship between *N*_area_ and *A*_net_ was observed (Fig. 8).

**Table 5:** Field measurements of leaf gas exchange parameters for the three species studied across two sampling periods and two soil types.

Species	*Acrostichum* (fern)				*Dalbergia* (N-fixer shrub)		*Laguncularia* (halophytic tree)			
Soil type	Martin Peña (Mp)		Saladar muck (Sm)		Saladar muck (Sm)		Martin Peña (Mp)		Saladar muck (Sm)	
Period	Wet	Dry	Wet	Dry	Wet	Dry	Wet	Dry	Wet	Dry
*A*_net_ µmol CO₂ m⁻² s⁻¹	3.53 ± 2.07	6.67 ± 1.38	8.27 ± 1.72	7.33 ± 2.7	5.74 ± 2.48	7.56 ± 1.73	10.44 ± 2.99	8.56 ± 2.34	12.59 ± 2.49	11.36 ± 2.07
*E* mmol H₂O m⁻² s⁻¹	0.39 ± 0.36	2.39 ± 1.41	1.62 ± 0.45	1.92 ± 0.70	0.42 ± 0.27	2.00 ± 0.45	2.33 ± 0.91	3.58 ± 1.48	3.12 ± 0.82	3.01 ± 0.57
*g_s_* mmol H₂O m⁻² s⁻¹	16.9 ± 16.8	87.40 ± 54.70	65.50 ± 24.50	118 ± 58.80	19.90 ± 14.4	120.00 ± 49.1	97.80 ± 41.00	162.00 ± 52	154.35 ± 54.50	217.00 ± 101.00
WUE µmol CO₂ mmol⁻¹ H₂O	8.96 ± 7.86	2.68 ± 1.91	5.37 ± 1.32	2.40 ± 1.50	15.1 ± 10.75	2.58 ± 0.72	5.05 ± 2.13	1.75 ± 0.54	4.17 ± 0.91	1.72 ± 1.87
*A*_n_/*g_s_* µmol CO₂ mmol⁻¹ H₂O	0.33 ± 0.42	0.12 ± 0.11	0.15 ± 0.06	0.07 ± 0.01	0.41 ± 0.38	0.07 ± 0.01	0.12 ± 0.05	0.06 ± 0.02	0.09 ± 0.02	0.06 ± 0.03
VPD kPa	2.28 ± 0.36	3.70 ± 0.83	2.62 ± 0.33	3.06 ± 0.65	2.20 ± 0.41	3.10 ± 0.64	2.46 ± 0.39	2.77 ± 0.44	2.15 ± 0.42	2.40 ± 0.68
PNUE µmol CO₂ m⁻² s⁻¹ per g N kg⁻¹	0.14 ± 0.09	0.19 ± 0.01	0.31 ± 0.06	0.19 ± 0.07	0.23 ± 0.09	0.30 ± 0.05	0.79 ± 0.27	0.56 ± 0.23	1.06 ± 0.27	0.61 ± 0.14

Parameters include net assimilation (*A_n_*), stomatal conductance (*g_s_*), WUE, intrinsic water use efficiency (*A_n_*/*g_s_*), vapour pressure deficit (VPD), and PNUE. Values represent mean ± standard deviation.

The field-measured photosynthesis data revealed species-specific and seasonal responses in leaf carbon assimilation (*A*_net_), transpiration rate (*E*), stomatal conductance (*g_s_*), and WUE. All measured variables were significantly influenced by hydrological conditions, with generally higher values during the wet period (June) compared to the dry period (February). *Acrostichum* (fern) exhibited strong period and soil-type variability, individuals in Saladar muck (Sm) soils demonstrated higher *A*_net_, *g_s_*, and lower WUE compared to Martin Peña (Mp) soils. *Dalbergia* (N-fixer shrub) and *Laguncularia* (halophytic tree) both showed pronounced responses to hydrological changes, with significant increases in foliar N and carbon concentrations during the wet period. In addition, WUE decreased in all species during the wet season due to higher transpiration rates associated with greater stomatal conductance.

## Discussion

Urban coastal wetlands experience strong environmental gradients and anthropogenic pressures that jointly shape plant physiological performance. Even under a shared mesoclimate, coexisting species differ in evolutionary history and ecological strategy, occupying distinct positions along the fast-slow resource continuum. These differences are expressed in intrinsic photosynthetic capacity, foliar structural traits, nutrient requirements, and tolerance to salinity and flooding. In addition to these natural drivers, anthropogenic disturbances, in particular heavy metal inputs can disrupt nutrient uptake and impair photosynthetic machinery and modify traits expression. In this study, we evaluated four hypotheses: three related to functional group differences in ecophysiological traits and a fourth testing the effect of anthropogenic metal inputs on photosynthetic performance across species.

Our results confirm that the three species representative of different plant functional groups (mangrove trees, understory ferns, and nitrogen-fixing shrubs) exhibit distinct ecophysiological responses to (i) spatial variability in soil elemental availability, (ii) hydrological conditions, and (iii) anthropogenic disturbances characteristic of tropical urban coastal wetlands as proposed in hypotheses 1–3. These ecophysiological responses are linked to the hydrological conditions of the wetland, exhibiting ecological strategies previously described that help explain species coexistence within the wetland. Rather than occupying the same ecological niche, these species share limiting resources through different functional traits.

Our first hypothesis was that *L. racemosa* (halophytic tree) would exhibit higher instantaneous and intrinsic WUE (higher A/*gs* and lower transpiration rates) as a strategy for coping with saline conditions. Our results confirm this, *Laguncularia* (halophytic tree) exhibited the highest light-saturated photosynthetic rates, quantum yield, and dark respiration rate, consistent with high photosynthetic capacity suited to high light environments typical of open canopy coastal systems (Fig. 6). It also exhibited the highest PNUE, indicating a higher adaptive behaviour under nutrient limited conditions (Lovelock and Feller 2003, Reef et al. 2010). It also exhibited greater WUE and salt tolerance than the other two species studied (Table 5). These findings are supported by findings that *Laguncularia* (halophytic tree) demonstrates greater salt tolerance and WUE than *Rhizophora mangle* in saline, nutrient-variable environments (Cardona-Olarte et al. 2013). Additionally, the combination of high leaf succulence and significant accumulation of Na, Mg, and Ca aligns with previously described halophytic adaptations (Fig. 8; Parida and Jha 2010). Notably, elevated concentrations of foliar Pb found in *Laguncularia* (halophytic tree) highlight its potential for heavy metal accumulation and phytoremediation, as previously suggested (Da Souza et al. 2014). These findings underline the species’ adaptive capacity to both saline and polluted environments, reinforcing its functional role in urban wetland resilience. Additionally, the observed reduction in photosynthetic rates in Mp soils (Fig. 7), where metal concentrations were highest, supports our hypothesis that anthropogenic inputs can decrease physiological performance. This consistency strengthens its role as a reliable bioindicator species, capable of reflecting the contamination status of estuarine and coastal ecosystems (Pimentel Victório et al. 2023).

The second hypothesis, we argued that *A. danaeifolium* (ferns) would display traits associated with shade tolerance and conservative resource use, including lower light-saturated photosynthetic rates and higher SLA. The results confirm this hypothesis, *Acrostichum* (fern) displayed the lowest light compensation point, low dark respiration rates, quantum yields, high SLA, and intermediate assimilation rates indicative of a shade-adapted photosynthetic strategy (Fig. 6, Table 4,Table 5; Medina et al. 1990), aligned with a strategy of efficient light capture in shaded understory environments (Evans and Poorter 2001, Fonini et al. 2017). Physiological tolerance to moderate salinity, supported by low K/Na ratios, greater succulence, and accumulation of Na and Ca, further underscores its physiological adaptation to variable salinity levels common in wetland understory microhabitats once established and supports its categorization as halotolerant. This is consistent with findings for *Acrostichum aureum* (Lugo et al. 2024). This combination of shade and salinity tolerance allows A. danaeifolium to occupy microhabitats that neither the mangrove *Laguncularia* nor the n-fixer shrub *D. ecastaphyllum* dominates, facilitating species coexistence.

The third hypothesis, that *D. ecastaphyllum* (N-fixer shrub) would exhibit higher nutrient-use efficiency due to nitrogen fixation was partially supported. *Dalbergia* (N-fixer shrub) exhibited higher N:P values (Fig. 4), consistently high WUE (especially during the dry period), and conservative stomatal behaviour (high *A*_net_/*g_s_* in the wet period), both traits indicative of an adaptive, resource-conserving strategy (Oram et al. 2021). However, its moderate PNUE and assimilation rates did not surpass *Laguncularia* (a halophytic tree), suggesting that nitrogen fixation alone does not guarantee higher productivity in this context. Its lower leaf Na content further supports a salt-exclusion mechanism. Although its nitrogen fixation capacity (Carmen Trujillo Pacheco et al. 2021) likely provides an advantage under nutrient-poor ecosystems (Reef et al. 2010), confirming this requires direct assessments of nodule activity and isotopic signatures (Fougnies et al. 2007). Ecologically, its high-WUE, resilience to salinity, and even to prolonged flooding after storms (Hernández et al. 2021) supports its role in stabilizing and recovering disturbed areas, making it a favourable species for restoration in coastal wetlands (Bohrer et al. 2008, Medina and Barboza 2006, Pereira and Mata 2014).

Lastly, we hypothesized that plants growing in anthropogenically influenced soils will exhibit lower photosynthetic performance because metal accumulation can lead to impairment of photosynthetic machinery and alter foliar elemental composition. This hypothesis was partially supported, as it was influenced by temporal dynamics. *Laguncularia* (halophytic tree) and *Acrostichum* (fern) had higher assimilation rates in less polluted soils (Saladar muck, Sm), but temporal differences were noted. Across all studied species, the significant spatial and temporal variation in soil elemental availability, particularly the elevated concentrations of Zn and Pb in Martin Peña (Mp) soils, reflects the influence of anthropogenic activities and historical land-use changes on soil chemistry (Table 3). These elements significantly influenced leaf chemistry and plant physiological responses, underscoring the effect of anthropogenic disturbances as described in previous studies and the relation between leaf and soil elemental composition (Martínez-Megías and Rico 2022, Newton et al. 2020).

Temporal dynamics strongly influenced plant ecophysiological parameters. During the wet period, increased water availability and nutrient solubility associated with elevated waterlogging conditions enhanced plant assimilation rates, stomatal conductance, and transpiration rates (Table 5). Conversely, during the dry period, reduced water availability led to increased WUE, most noticeable in *Dalbergia* (N-fixer shrub). These findings emphasize the role of hydrological conditions, mainly water levels and to a lesser extent the influence of moderate soil salinity, influencing species-specific responses along the terrestrial–marine continuum and their potential implications for plant performance under changing environmental conditions in coastal wetlands (Hogan et al. 2022).

Overall, the three species express different resource-use strategies, ranging from fast (*Laguncularia*), conservative (*Acrostichum*), and intermediate (*Dalbergia*) strategies, reducing direct competition and coexisting under shared environmental conditions. Their ecophysiological differences reflect long-term evolutionary specialization and result in the partitioning of resources and acquisition strategies. The individual roles contribute to the resilience and the processes and functions of novel urban coastal wetlands under ongoing anthropogenic pressures, whose structure and function are shaped by legacy infrastructure, human pressures, and extreme disturbances, rather than returning to historical baselines (Lugo 2020).

## Conclusions

This study shows the distinct ecophysiological adaptations of urban coastal wetland plants in response to anthropogenic disturbances, soil element variability, and fluctuating hydrological conditions. In Cienaga Las Cucharillas wetland, vegetation appears to be opportunistic, establishing wherever space and resources become available, and resilient to multiple interacting stressors, both anthropogenic and climate-related extremes, such as Hurricane Maria (2017). Overall, our results point to an ecosystem that is functioning under chronic stress, expressed in a species-specific trait variability and temporal shifts, suggesting adjustment to current stressors. In that sense, the wetland seems to be coping in the short term, but this also could suggest vulnerability to future acute disturbances, as the trade-offs might constrain resilience. Understanding these adaptive strategies is essential for predicting plant community resilience and informing restoration practices in increasingly urbanized coastal landscapes. Effective wetland management and restoration strategies should incorporate these species-specific adaptive traits and physiological responses when selecting vegetation for targeted ecological functions. Future research should explore long-term ecological effects of heavy metal accumulation, assess the role of nitrogen-use efficiency mechanisms in nitrogen-fixing shrubs on wetland biogeochemical dynamics, and evaluate ecosystem-level responses to ongoing environmental changes and test if the observed trait plasticity translates into sustained productivity at a longer time scale. These efforts will be crucial to strengthening evidence-based wetland management and conservation strategies.

## Supplementary Material

plag006_Supplementary_Data

## Data Availability

Raw data are available online through TRY Plant Trait Database File Archive (https://www.try-db.org) under DOI 10.17871/TRY.115 and 10.17871/TRY.116
