# Supplementary figures and images for: Functional traits and elemental uptake in urban coastal wetland plants under variable hydrology and edaphic conditions
Source: AoB Plants. 2026 Feb 10;18(1):plag006. doi: 10.1093/aobpla/plag006 (PMC12948549; doi:10.1093/aobpla/plag006)

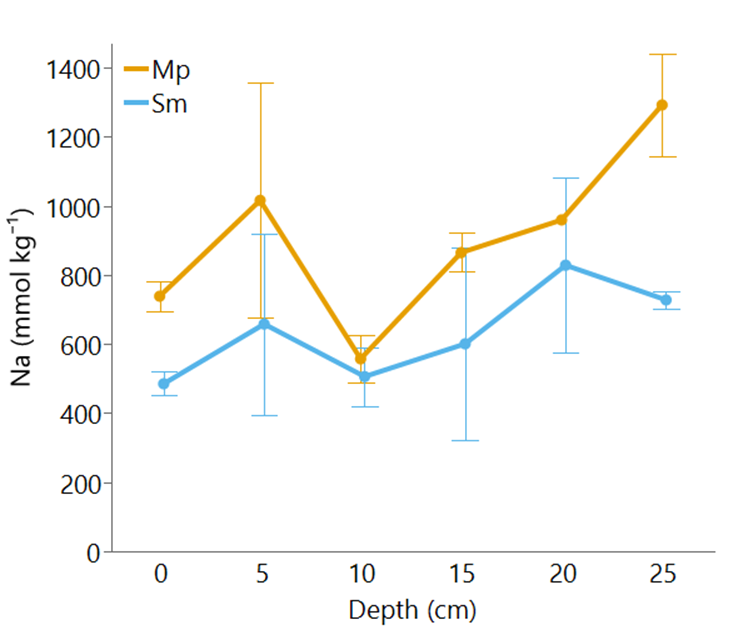

Supplement: plag006_Supplementary_Data [file plag006_supplementary_data.zip › FigureS1.png]

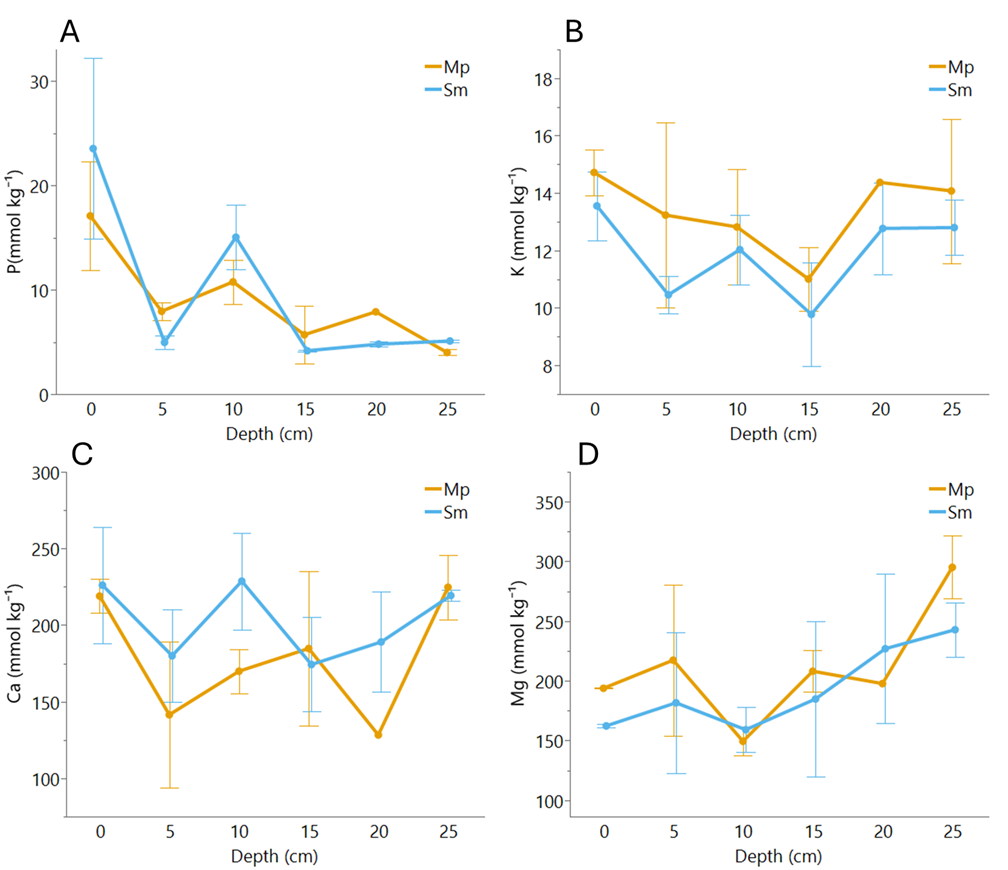

Supplement: plag006_Supplementary_Data [file plag006_supplementary_data.zip › FigureS2.png]

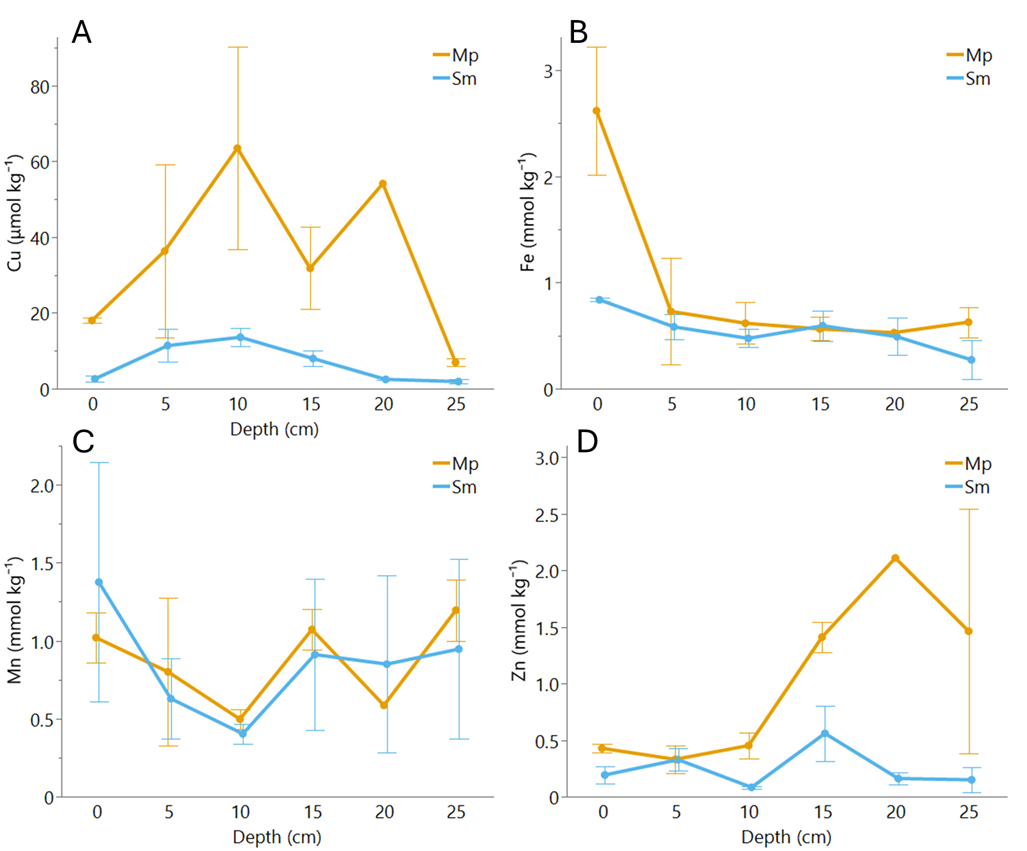

Supplement: plag006_Supplementary_Data [file plag006_supplementary_data.zip › FigureS3.png]

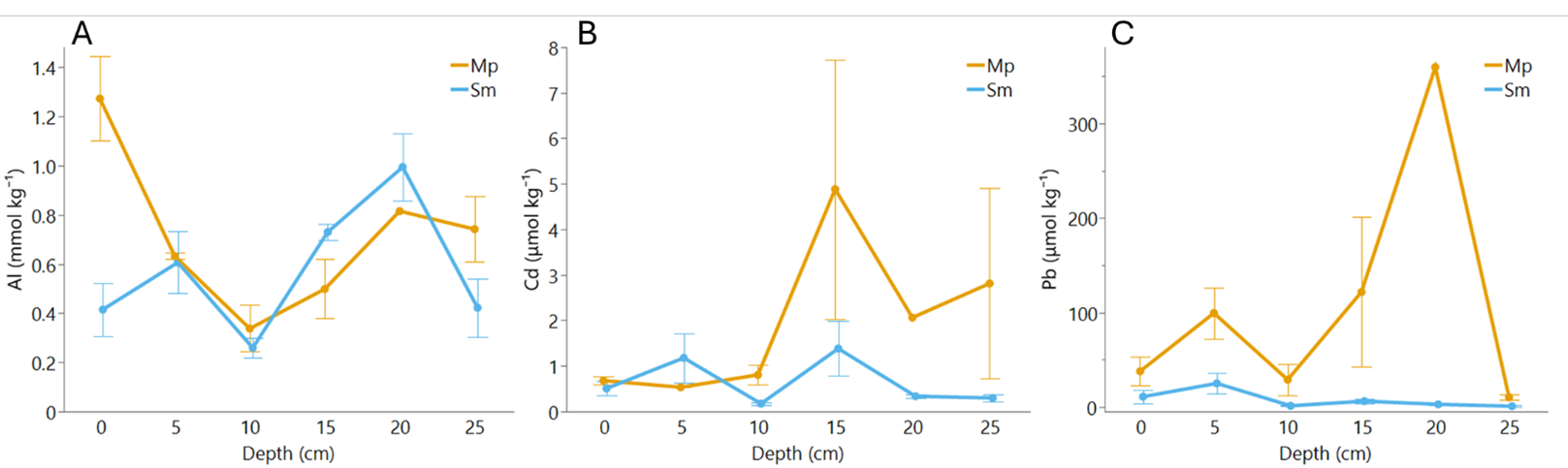

Supplement: plag006_Supplementary_Data [file plag006_supplementary_data.zip › FigureS4.png]
